# Association Between Joint Pain and Cancer in 8.45 Million Korean Adults: Insights from a National Cross-Sectional Study

**DOI:** 10.3390/jcm14051478

**Published:** 2025-02-22

**Authors:** Taewook Kim

**Affiliations:** Department of Orthopedic Surgery, Seoul National University Hospital, Seoul 03080, Republic of Korea; ray0601@snu.ac.kr

**Keywords:** joint pain, osteoarthritis, Kellgren–Lawrence grading, gastric cancer, liver cancer, colorectal cancer, breast cancer, cervical cancer, lung cancer, thyroid cancer

## Abstract

**Background:** Joint pain, a multifactorial musculoskeletal symptom, is rising globally due to an aging population. Simultaneously, cancer is increasingly considered a chronic condition with growing prevalence and improved survival rates, similar to hypertension and diabetes. Although the association between chronic diseases such as diabetes and joint pain has been well studied, the relationship between cancer and joint pain remains underexplored, especially as cancer’s chronic disease status evolves. **Methods:** This study analyzed data from the Korean National Health and Nutrition Examination Survey (KNHANES V) to investigate associations between cancer and joint pain in 8,451,047 individuals, representing Koreans over 50. Descriptive analyses identified demographic characteristics and disparities in joint pain prevalence by age and sex. Multivariate logistic regression analyzed seven common cancers in relation to spine, hip, and knee pain, adjusting for various factors and the Kellgren–Lawrence radiographic grade to pinpoint cancers significantly associated with each joint pain type. **Results:** Analysis demonstrated significant associations between certain cancers and joint pain. Back pain was linked to gastric, liver, cervical, and lung cancers; hip pain to breast and thyroid cancers; and knee pain to liver cancer. These findings underline complex relationships that suggest further investigation is needed to clarify specific cancer-related joint pain mechanisms. **Conclusions:** Descriptive and regression analyses highlighted essential demographic factors and significant associations between certain cancers and joint pain types. These insights enhance understanding of cancer’s chronic impact on joint pain and underscore the need for further research to refine these associations.

## 1. Introduction

Joint pain is a chronic symptom with heterogeneous and multifactorial characteristics that affect various joints across the body and exhibit diverse progression patterns with age [1]. The increasing incidence of joint pain has emerged as a notable issue in the context of global aging. Despite considerable international variations in the prevalence, incidence, and years lived with disability due to joint pain, the burden associated with joint pain is increasing in most countries [2]. Moreover, this trend is expected to persist because of higher life expectancies and the aging of the global population.

Recent research on the global burden of disease has revealed the significant impact of cancer worldwide [3]. Unlike the improvements observed in other non-communicable diseases, a worsening trend has been identified in the burden of cancer [4]. In Korea, cancer incidence rates are projected to decrease for all types of cancer. Cancer mortality rates have shown a declining trend since 2002, with an annual decrease of 2.8% [5]. However, as survival rates improve, cancer is increasingly regarded as a chronic condition, similar to hypertension and diabetes. This shift has led to a growing focus on understanding cancer’s long-term impact on comorbid conditions and quality of life [6].

Previous studies have extensively examined the connections between chronic diseases, such as dyslipidemia and hypertension, and joint pain, finding clear associations. However, despite the chronic disease status of many cancers, limited research has explored the relationship between cancer and joint pain, leaving an important gap in our understanding of the full impact of cancer on musculoskeletal health [7,8].

To address this knowledge gap, this study aimed to elucidate the relationship between cancer and joint pain by examining how various cancers may contribute to joint pain prevalence among Koreans aged 50 and over. Our analysis focused on seven prevalent cancers (gastric, liver, colorectal, breast, cervical, lung, and thyroid cancer) and three joint pain types (spine, hip, and knee) to identify specific cancer-pain associations. This comprehensive approach included a multivariate logistic regression analysis of joint pain in patients with multiple cancer diagnoses, using data from the Korean National Health and Nutrition Examination Survey (KNHANES), a nationally representative cross-sectional study. Due to the use of sampling weights, this study can be generalized to reflect the entire Korean population (N = 8,451,047).

By identifying specific associations between cancer types and joint pain, this study aims to provide valuable insights into the chronic effects of cancer on joint pain. Although establishing causality is difficult due to the cross-sectional design, our study aims to highlight the potential indication of a link between cancer and joint pain in cancer survivors.

## 2. Materials and Methods

### 2.1. Data Source

This study recruited participants from the fifth iteration of the Korea National Health and Nutrition Examination Survey (KNHANES V), which was conducted between 2010 and 2011. The Korea National Health and Nutrition Examination Survey (KNHANES), established in 1998, is a cross-sectional study aimed at evaluating the health status of the Korean population. It serves as a valuable resource across multiple research disciplines, including medicine and social sciences [9,10,11]. Administered by the Korea Disease Control and Prevention Agency, the survey includes approximately 10,000 participants selected from 3840 households across 192 regions of South Korea each year. A key feature of the KNHANES is its precise calculation of sampling weights, which accounts for variables such as household composition across generations, geographic regions, and age groups. The KNHANES is considered representative of the entire Korean population (N = 46,286,503) after adjustment using a two-stage sampling method that includes stratification, clustering, and weighting. The meticulous implementation of sampling weights guarantees that the derived estimates reliably represent the characteristics of the Korean population. Additional information regarding the methodology for calculating these weights can be found in the relevant literature [12].

**Figure 1:**
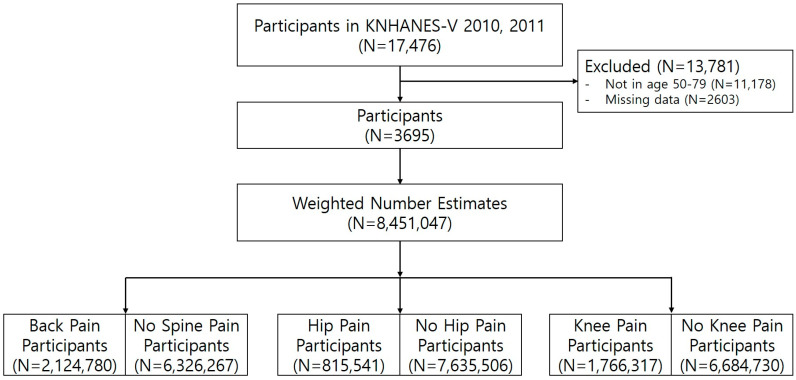
Study design that ensures the representation of all Koreans through the use of complex sampling weights.

Figure 1 depicts the process of participant selection, starting with 17,476 individuals from KNHANES 2010–2011. Participants with missing or inaccurate data, those who did not undergo radiography (n = 2603), and those aged over 80 or below 50 years (n = 11,178) were excluded. Ultimately, data from 3695 participants aged 50–79 years were analyzed. The study focused on individuals aged 50 to 79, as the KNHANES survey only asked about joint pain for those 50 and older, while respondents aged 80 and above were grouped into a single category (80 years and older) in the reports. Consequently, we concluded that focusing our analysis on the 50–79 age group was the most suitable approach. Data from 2010–2011 were utilized, as this was the period when KNHANES incorporated X-ray imaging. While KNHANES surveys have continued beyond this timeframe, X-ray data were omitted in subsequent surveys due to factors such as budget limitations. To ensure the representation of all Koreans, complex sampling weights were employed, yielding a weighted number estimate of N = 8,451,047. The use of sampling weights guaranteed that the calculated estimates truly reflected the Korean population. The weighted number estimate was further stratified based on the experience of joint pain such as back, hip, and knee pain.

### 2.2. Characteristics of KNHANES Database

KNHANES comprises nine surveys, and for our study, we used health interviews, health examination data, and dual-energy X-ray absorptiometry (DEXA) for body composition [13]. Health interviews, encompassing epidemiological factors, were conducted using questionnaires, while health examination data, including medical metrics such as height and weight, were gathered by national organizations. Dual-energy X-ray absorptiometry (DEXA), employed for diagnosing osteoporosis, was administered at mobile examination units.

#### 2.2.1. Epidemiological Factors

Occupation was categorized into two groups: white-collar jobs, including office and professional roles, and blue-collar jobs, representing manufacturing and labor roles. The residential areas were categorized based on location, distinguishing between residents living in cities and those residing in rural regions of Korea.

#### 2.2.2. Medical Factors

In this study, both subjective pain perception and objective radiographic grades were used to analyze joint pain. Radiographs were evaluated using the Kellgren–Lawrence grading system [13]. X-ray grading was independently performed by two radiologists, and in cases of a one-grade discrepancy, the higher grade was used for analysis. For discrepancies exceeding one grade, a third radiologist conducted a thorough review, and the grade determined by this third assessment was adopted. Following established research practices, Kellgren–Lawrence grades 2 to 4 were classified as indicative of radiographic findings, while grades 0 and 1 were considered normal. As the Kellgren–Lawrence grading system is primarily based on X-ray changes associated with osteoarthritis (OA), cases identified as high grades in our study were categorized as OA. The presence of joint pain was ascertained through a survey that asked participants about their pain experiences. Joint pain was specifically defined as discomfort persisting for a minimum of 30 days within the prior three months. Participants who answered “yes” to this question in the survey were classified as experiencing joint pain.

The KNHANES data only assigned grades to the most affected joint. For instance, participants were instructed to respond based on the more painful joint between the two hip joints or knee joints. Similarly, for spine pain, they were asked to report based on the most painful area of the spine, such as the thoracic, lumbar, or sacral regions

Depression, defined as a depressive mood lasting more than two weeks, is considered a risk factor. Additionally, osteoporosis was identified as a binary variable based on participants’ affirmative responses to the question, “Have you ever been diagnosed with osteoporosis by a doctor?” Also, individuals with DEXA scan T-scores of −2.5 or lower were included under the diagnostic criteria for osteoporosis.

### 2.3. Statistical Analysis

The baseline characteristics of male and female participants were analyzed separately to explore potential sex-based differences. Participants were also categorized into three age groups (50–59, 60–69, and 70–79 years) for comparison. Continuous variables, such as age, were presented as mean ± standard deviation and evaluated using the Student’s *t*-test. Categorical variables, including radiographic findings, were displayed as percentages and counts and compared using the chi-squared test. Normality tests were applied to all variables, and those that did not meet the assumption of normality were analyzed with non-parametric methods, specifically the Mann–Whitney U test.

To determine whether the cancer diagnosis can serve as a related factor for musculoskeletal pain, a multivariate logistic regression analysis focusing on back, hip, and knee pain was performed [14]. The analysis included covariates such as age, sex, body mass index (BMI), menopause, depression, osteoporosis, and various factors, which are commonly utilized in joint pain research [15,16,17]. Odds ratios (ORs) with 95% confidence intervals (CIs) were calculated to quantify the relationships. Additionally, the variance inflation factor (VIF) was analyzed to check for multicollinearity among the variables [18].

## 3. Results

### 3.1. Descriptive Analysis

An analytical overview highlighted key characteristics and sex-based disparities (Table 1). Within the Korean population aged 50–79, 2,124,780 individuals (25.1%) had back pain, and 2,384,589 (28.2%) showed abnormalities in spine radiographs. Hip pain affected 815,541 (9.7%), with 78,590 (0.9%) displaying hip radiographic findings. Additionally, knee pain was reported in 1,766,317 (20.9%), and knee radiographic abnormalities were observed in 2,881,843 (34.1%).

Female patients were generally older, had a higher BMI, were less engaged in manual labor, and more frequently resided in rural locations. Female patients experienced more joint pain and showed greater radiographic severity, although severe hip radiographic findings were more prevalent in male patients. Conditions such as depression, osteoporosis and colorectal, breast, cervical, and thyroid cancers were predominantly associated with female sex, whereas stomach, liver, and lung cancers were more linked to male sex.

This study also examined the age distribution of the participants, focusing on three age brackets: 50–59, 60–69, and 70–79 years, as detailed in Table 2. As expected, aging was associated with a higher incidence of musculoskeletal issues, including back pain, hip pain, knee pain, and radiographic OA of the spine and knee. However, radiographic hip OA was not clearly associated with age. A decline in engagement in blue-collar labor and an increase in residence in rural areas were also observed with aging. The relationship between aging and cancer incidence was found to vary, indicating that the effect of aging on cancer prevalence depends on the type of cancer.

### 3.2. Cancer as a Related Factor for Back Pain

Using multivariate logistic regression, this study examined the related factors for back pain (Table 3). Back pain was associated with gastric, liver, cervical, and lung cancers, whereas colorectal, breast, and thyroid cancers were associated with a lower prevalence of back pain. Factors such as aging, female sex, menstruation, depression, and osteoporosis were correlated with an increased sensation of back pain. Employment in blue-collar jobs and residence in rural areas were also identified as factors associated with back pain. The VIF for all factors was less than 10, indicating no multicollinearity.

### 3.3. Cancer as a Related Factor for Hip Pain

In the examination of the related factors for hip pain (Table 4), hip pain was associated with breast and thyroid cancers, whereas gastric, colorectal, cervical, and lung cancers were linked to a reduced prevalence of hip pain. Factors such as aging, female sex, high BMI, menstruation, depression, and osteoporosis were correlated with an increased sensation of hip pain. Furthermore, white-collar employment and residence in rural areas were recognized as factors associated with hip pain. The VIF for all evaluated factors was less than 10, suggesting the absence of multicollinearity.

### 3.4. Cancer as a Related Factor for Knee Pain

This study also investigated the related factors associated with knee pain (Table 5). The findings revealed a connection between knee pain and liver cancer, whereas colorectal, breast, cervical, lung, and thyroid cancers were associated with a reduced prevalence of knee pain. Factors such as aging, female sex, high BMI, menopause, depression, and osteoporosis were associated with an increased likelihood of knee pain. Additionally, working in occupations requiring manual labor and residing in rural settings were identified as contributors to knee pain. The VIF for the analyzed factors was less than 10, indicating no significant multicollinearity.

## 4. Discussion

This study aimed to characterize the relationship between cancer and joint pain in the entire Korean population aged 50–79 years using data from the Korea National Health and Nutrition Examination Survey (KNHANES V). The prevalence of joint pain, specifically back pain (25.1%), hip pain (9.7%), and knee pain (20.9%), was determined, and significant radiographic abnormalities indicating back, hip, and knee OA were noted in 28.2%, 0.9%, and 34.1% of Korean participants, respectively.

One noteworthy finding was the association between joint pain and various demographic and clinical characteristics. Females and older individuals experienced more severe joint pain, which is consistent with prior research [19]. The inverse relationship between BMI and knee pain, in contrast to the positive correlation between BMI and hip or back pain, underscores the complex interplay between obesity and joint pain. Previous studies have shown that a high BMI increases the load on the joints, leading to greater pain [20]. However, the cross-sectional nature of this study precluded the establishment of a causal relationship. In other words, the findings may imply that individuals with obesity who engage in less physical activity might experience less joint pain due to the reduced usage of their joints. In clinical settings, when patients with a high BMI are evaluated, it is important not to always assume a higher likelihood of joint pain but also to consider the possibility of less joint pain due to lower physical activity and reduced joint load.

Engagement in blue-collar occupations has been linked to increased instances of spinal and knee pain, whereas residing in rural areas has been associated with a heightened perception of pain. This connection has been extensively examined in previous studies [21]. The high physical demands of blue-collar occupations have been identified to contribute to the increased prevalence of joint pain. KNHANES classified occupations into seven categories: (1) managers, professionals, and related occupations; (2) office workers; (3) service and sales workers; (4) skilled agricultural, forestry, and fishery workers; (5) craft and machine operators and assemblers; (6) elementary occupations; and (7) unemployed, students, and homemakers. In our study, we categorized groups 1–3 as white-collar occupations, aligning with previous research that has adopted similar classifications. While it would have been ideal to incorporate more precise quantifications of physical activity and occupational exposure, the KNHANES dataset does not provide detailed information on these factors. We acknowledge that a more refined analysis considering physical activity levels could further enhance our understanding of occupational risk factors for joint pain. This limitation has been addressed in Section 4. Additionally, limited access to medical facilities in rural areas has been recognized as a factor contributing to the increased prevalence of musculoskeletal conditions [22,23].

An investigation into the correlation between cancer and joint pain revealed complex results. This analysis, utilizing multivariate logistic regression and adjusting for factors such as age, sex, BMI, menopause, depression, osteoporosis, and radiographic grade, identified specific associations between certain types of cancers and joint pain. For instance, liver cancer was positively correlated with back and knee pain. In contrast, breast and thyroid cancer are associated with hip pain.

Specific cancer-related factors are beneficial when analyzing the correlation between cancer and joint pain. One notable example is breast cancer, for which estrogen is a key risk factor [24]. Intriguingly, estrogen has also been implicated as a risk factor for osteoporosis and bone joint-related disorders. Estrogen, a hormone chiefly involved in female reproductive health, plays a crucial role in maintaining bone density and regulating bone metabolism. A decline in estrogen levels, particularly during menopause, can lead to bone loss, osteoporosis, and an elevated risk of bone and joint issues [25]. Women with breast cancer may experience fluctuations in estrogen levels due to the cancer itself or its treatment, potentially exerting adverse effects on bone health and joint function.

The role of inflammation has been considered in different perspectives on the relationship between cancer and joint pain [26]. Inflammation and inflammatory cells are widely acknowledged to contribute to the development of cancer. OA leads to a chronic inflammatory state, which, in turn, increases the risk of cancer [27]. However, because OA-related inflammation is localized around the joints, this theory cannot be readily applied to cancers located far from the joints, such as liver and stomach cancers. Furthermore, the exact mechanisms by which inflammation associated with OA is linked to specific cancer risks at particular sites remain elusive [28]. While OA results in higher levels of circulating byproducts from cartilage, bone, or synovial damage, no conclusive evidence has been obtained to suggest that these byproducts either promote or protect against cancer [29,30]. If this relationship is interpreted solely in terms of inflammation, it would imply that all forms of joint pain should present with uniform severity. However, the inconsistent results in this study reflect the inherent complexities of such an interpretation.

The association between cancer and joint pain can also be examined in terms of the medications used to treat joint pain [31], which may be associated with the observed cancer risks. Approximately 40–55% of patients with joint pain regularly take non-aspirin non-steroidal anti-inflammatory drugs (NSAIDs), while 10–20% regularly take acetaminophen [32]. The consumption of non-aspirin NSAIDs has been linked to a decreased risk of developing colorectal, esophageal, and stomach cancers as well as colonic adenomas [33]. Evidence linking NSAID use with a reduced risk of hepatobiliary, pancreatic, and oropharyngeal cancers remains scarce [34]. Previous studies on the use of NSAIDs and their impact on lung cancer risk have produced mixed results. However, longer periods of NSAID usage have been frequently shown to be associated with a protective effect against lung cancer [35]. Additionally, a reduced risk of postmenopausal breast cancer has been observed in women who consume non-aspirin NSAIDs [36].

Chemotherapy is known for its potential to induce joint damage. Chemotherapy is associated with varying levels of inflammatory response, and previous studies have indicated a correlation between joint pain and the use of chemotherapy or hormonal therapy [37]. However, the diversity of chemotherapeutic drugs used for distinct cancers poses a challenge for research and warrants further discussion.

The association with psychological factors should also be considered. In our study, we found that experiencing depressive symptoms for more than 14 days was significantly associated with an increased prevalence of back pain, hip pain, and knee pain. Multiple studies have shown that depression can exacerbate psychosomatic pain, thereby contributing to joint pain [38,39]. Therefore, it is reasonable to suggest that the experience of pain in cancer patients may also be influenced by psychiatric aspects.

In the context of cancer-related joint pain, alterations in cartilage biomechanics may contribute to symptom severity. Cancer patients often experience inflammatory responses, systemic metabolic changes, and potential side effects from cancer treatments, all of which may exacerbate joint deterioration and pain [40,41].

Although this study provides valuable insights into the less-explored associations between cancer and joint pain, it has several limitations that require consideration. First, unlike other national cohort data that collect data longitudinally [42], the cross-sectional nature of KNHANES V limits the ability to establish causality. In other words, it is difficult to determine whether pain develops after cancer or if cancer arises following the onset of pain. While it is reasonable to interpret that joint pain results from cancer, this interpretation cannot be statistically validated due to the inherent limitations of a cross-sectional study design. This limitation highlights the need for further longitudinal research to better understand the temporal relationship between cancer and joint pain.

Second, the lack of detailed information about cancer, such as staging, chemotherapy or radiation therapy history, and the duration since diagnosis, limited our understanding of the relationship between cancer and joint pain. Furthermore, the absence of details regarding bone metastasis, which is known to cause severe bone pain and reduced physical activity, may pose a limitation in our assessment of joint pain. This limitation arises because the KNHANES survey does not ask for information about bone metastasis, highlighting a constraint in our study design. Considering that spinal metastasis and metastatic cancer in the femur can lead to severe pain, such as impending fractures, this represents a significant gap in our analysis. We hope that future studies will address this critical area of research.

Moreover, self-reported data collected through KNHANES questionnaires may introduce biases, including recall bias. The KNHANES survey asked whether participants experienced pain in the affected joint but did not specify the circumstances, timing, or characteristics of the pain. Additionally, there was no objective or quantitative assessment of pain intensity. Such limitations have been observed in several studies conducted using KNHANES data [10,38,43]. Future studies should incorporate validated pain assessment tools, such as the Visual Analog Scale (VAS) or the Western Ontario and McMaster Universities Osteoarthritis Index (WOMAC), to improve the objectivity and reproducibility of pain measurements. In addition, while our study focused solely on depressive symptoms, a cancer diagnosis is often a significant life event. Therefore, future research should explore other psychiatric conditions, such as anxiety and psychotic disorders, to better understand their potential impact on pain perception in cancer patients. Furthermore, the survey did not address bone-forming nutrients like Vitamin D or consider the history of painkiller use or recent trauma, which could have offered deeper insights into joint pain and X-ray results.

Additionally, the lack of data about previous joint conditions, such as previous joint surgery, a history of septic arthritis, or the presence of joint arthroplasty, restricted our ability to provide comprehensive information on joint pain and X-ray findings

Our study considered KL grades 2 to 4 as a single radiographic OA group, following previous research [38,39,43]. Since our study aims to explore the relationship between joint pain and cancer as a chronic disease, this study found limited rationale for focusing exclusively on high-grade levels based on X-ray findings. However, it is typical to consider KL grade 2 as mild osteoarthritis, characterized by minor osteophyte formation and minimal joint space narrowing, while KL grades 3 and 4 generally indicate more advanced OA. Therefore, grouping grades 3 and 4 alone could also be meaningful. To maintain consistency with previous studies based on KNHANES data, we adopted the same classification by grouping KL grade 2 and above as a single category. However, future studies focusing specifically on severe KL grades 3 or 4 would likely provide further valuable insights.

Another limitation of our study is that we could not analyze pain quantitatively in greater detail. Specifically, the lack of detail on pain intensity and frequency may affect the interpretation of the association between cancer and joint pain. Moreover, the age range of participants was restricted. We opted to include only individuals aged 50 and above because KNHANES conducted X-ray examinations and assessed joint pain solely for this age group. Furthermore, we set the upper age limit below 80 years, as individuals aged 80 and older were grouped together in the survey, making precise evaluations difficult. This decision was based on the KNHANES dataset structure, which categorizes individuals aged 80 and older as a single group due to the small sample size. Accordingly, we followed this categorization in our analysis. While analyzing all age groups might have provided more comprehensive results, the age restrictions in this study were necessary given the available data.

Finally, another limitation of our study is the possibility that the associations observed between cancer and joint pain may have occurred by chance. Although multivariate logistic regression analysis was employed to adjust for confounding factors such as age, sex, BMI, and radiographic grades, the inherent limitations of cross-sectional data and the absence of certain critical variables, such as detailed cancer characteristics (e.g., cancer stage, treatment history, or metastasis status), might have influenced our findings. As a result, the statistical associations identified in this study should be interpreted with caution.

However, conducting a well-designed prospective study with a similarly large sample size of 8.45 million individuals, which accounts for all potential factors, presents significant logistical and practical challenges. Despite these limitations, this study is meaningful, as it is the first to describe the association between cancer and joint pain at a population level. We hope that future studies with more rigorous designs, encompassing individuals of all age groups, will further investigate this relationship.

In summary, the findings of this study highlight the intricate relationship between cancer and joint pain in the Korean population. These findings underscore the need for comprehensive assessments encompassing not only demographic and clinical factors but also specific characteristics related to cancer. Future longitudinal studies that incorporate additional cancer-related parameters and observational data collected over an extended period to establish causal relationships may provide a more detailed understanding of this complex interplay, ultimately facilitating improved management and targeted interventions for individuals experiencing joint pain due to cancer.

## 5. Conclusions

The descriptive analyses in this study emphasized key demographic characteristics, particularly highlighting the prevalence of back, hip, and knee pain along with abnormal radiographic findings. Through multivariate logistic regression analyses, significant associations were identified, providing a clearer understanding of the complex relationships between specific cancer types and joint pain.

In summary, this study addresses an underexplored area by examining the link between cancer and joint pain in an aging population. Utilizing the nationally representative KNHANES dataset, the findings offer valuable insights into this intricate relationship. However, as a cross-sectional study, it cannot establish causality. Additionally, limited information on cancer specifics, such as stage and prior treatment history, presents a notable limitation. Future research should aim to address these gaps to further clarify the cancer–joint pain connection.

## Figures and Tables

**Table 1:** Descriptive analysis of participants categorized by sex.

	Total (N = 8,451,047)	Male (n = 3,976,209)	Female (n = 4,474,838)
Age (year)	61.0 ± 8.2	60.4 ± 7.9 *	61.4 ± 8.5 *
BMI	24.1 ± 3.1	23.8 ± 2.9 *	24.3 ± 3.2 *
Occupation (blue-collar)	46.1%	57.5% *	35.9% *
Residence (living in a rural area)	29.6%	29.5% *	29.7% *
Menopause	48.4%	0% *	91.5% *
Depressive mood (>14 days)	15.3% *	10.5% *	19.5% *
Diagnosis of osteoporosis	9.4%	1.3% *	16.5% *
Diagnosis of gastric cancer	0.8%	1.1% *	0.7% *
Diagnosis of liver cancer	0.2%	0.4% *	0.1% *
Diagnosis of colorectal cancer	0.5%	0.4% *	0.5% *
Diagnosis of breast cancer	0.8%	0% *	1.6% *
Diagnosis of cervical cancer	0.5%	0% *	0.9% *
Diagnosis of lung cancer	0.2%	0.3% *	0.1% *
Diagnosis of thyroid cancer	0.7%	0.0%	1.2%
Back pain	25.1%	14.1% *	34.9% *
Radiographic spine OA	28.2%	24.6% *	31.5% *
Hip pain	9.7%	5.4% *	13.4% *
Radiographic hip OA	0.9%	1.5% *	0.4% *
Knee pain	20.9%	11.2% *	29.5% *
Radiographic knee OA	34.1%	24.4% *	42.7% *

* *p* < 0.05; BMI, body mass index; radiographic OA, radiological findings indicating more than moderate OA based on the Kellgren–Lawrence grading system.

**Table 2:** Descriptive analysis of participants by age groups.

	Aged 50–59 Years (N = 4,324,730)	Aged 60–69 Years (n = 2,505,196)	Aged 70–79 Years (n = 1,621,121)
Age (year)	54.1 ± 2.8 *	64.4 ± 2.9 *	73.9 ± 2.8 *
Sex	50.8% *	52.0% *	60.2% *
BMI	24.1 ± 2.9 *	24.2 ± 3.1 *	23.8 ± 3.3 *
Occupation (blue-collar)	51.5% *	46.9% *	30.3% *
Residence (living in rural area)	25.9% *	30.9% *	37.5% *
Menopause	41.9% *	52.0% *	60.2% *
Depressive mood (>14 days)	16.2% *	14.1% *	14.6% *
Diagnosis of osteoporosis	4.8% *	12.5% *	16.8% *
Diagnosis of gastric cancer	0.3% *	1.5% *	1.4% *
Diagnosis of liver cancer	0.2% *	0.1% *	0.6% *
Diagnosis of colorectal cancer	0.1% *	0.8% *	1.1% *
Diagnosis of breast cancer	0.9% *	0.8% *	0.8% *
Diagnosis of cervical cancer	0.4% *	0.8% *	0.3% *
Diagnosis of lung cancer	0.1% *	0.1% *	0.4% *
Diagnosis of thyroid cancer	0.8% *	0.3% *	0.9% *
Back pain	17.9% *	28.8% *	38.7% *
Radiographic spine OA	15.8% *	31.5% *	56.4% *
Hip pain	6.7% *	10.5% *	16.3% *
Radiographic hip OA	1.1% *	0.5% *	1.2% *
Knee pain	13.0% *	26.0% *	34.2% *
Radiographic knee OA	19.5% *	43.7% *	58.2% *

* indicates *p* < 0.05; sex, female; BMI, body mass index; radiographic OA, radiological findings indicating more than moderate osteoarthritis based on the Kellgren–Lawrence grading system.

**Table 3:** Multivariate logistic regression for back pain in relation to diagnosis of cancer.

Characteristics	Coefficient	Standard Error	Odds Ratio (95% CI)	*p*-Value
Sex	1.204	0.004	3.333 (3.305–3.362)	<0.001
Age	0.036	0.00001	1.037 (1.036–1.037)	<0.001
BMI	−0.00006	0.00002	0.999 (0.999–1.000)	0.828
Occupation	0.048	0.002	1.049 (1.045–1.053)	<0.001
Residence	0.824	0.001	2.280 (2.272–2.289)	<0.001
Menopause	−0.184	0.004	0.831 (0.824–0.838)	<0.001
Depression	0.661	0.002	1.937 (1.928–1.945)	<0.001
Osteoporosis	0.771	0.002	2.162 (2.150–2.173)	<0.001
Gastric cancer	0.461	0.008	1.586 (1.560–1.613)	<0.001
Liver cancer	0.884	0.016	2.420 (2.344–2.499)	<0.001
Colorectal cancer	−0.554	0.013	0.552 (0.559–0.589)	<0.001
Breast cancer	−0.350	0.009	0.704 (0.692–0.717)	<0.001
Cervical cancer	0.037	0.011	1.038 (1.016–1.061)	<0.001
Lung cancer	0.638	0.019	1.893 (1.823–1.965)	<0.001
Thyroid cancer	−0.172	0.009	0.841 (0.825–0.857)	<0.001
Radiographic spine OA	0.559	0.002	1.749 (1.743–1.756)	<0.001
Constant	−4.660			<0.001

CI, confidence interval; sex, female; BMI, body mass index; occupation, blue-collar job; residence, living in a rural area; depression, feeling depressed for more than 2 weeks; radiographic spine OA, radiological findings indicating more than moderate osteoarthritis of the spine based on the Kellgren–Lawrence grading system.

**Table 4:** Multivariate logistic regression analysis for hip pain in relation to diagnosis of cancer.

Characteristics	Coefficient	Standard Error	Odds Ratio (95% CI)	*p*-Value
Sex	0.952	0.006	2.592 (2.560–2.625)	<0.001
Age	0.045	0.0001	1.046 (1.045–1.046)	<0.001
BMI	0.001	0.0003	1.001 (1.000–1.002)	<0.001
Occupation	−0.110	0.002	0.895 (0.890–0.903)	<0.001
Residence	0.287	0.003	1.332 (1.325–1.339)	<0.001
Menopause	−0.230	0.006	0.794 (0.784–0.803)	<0.001
Depression	0.511	0.009	1.666 (1.657–1.676)	<0.001
Osteoporosis	0.782	0.003	2.185 (2.172–2.199)	<0.001
Gastric cancer	−0.168	0.012	0.844 (0.824–0.866)	<0.001
Liver cancer	−12.90	9.99	0.000002 (0–680.979)	0.193
Colorectal cancer	−13.53	6.76	0.000001 (0–0.759)	<0.05
Breast cancer	0.348	0.010	1.416 (1.388–1.444)	<0.001
Cervical cancer	−0.577	0.018	0.561 (0.542–0.581)	<0.001
Lung cancer	−0.628	0.032	0.535 (0.503–0.570)	<0.001
Thyroid cancer	0.817	0.011	2.263 (2.217–2.231)	<0.001
Radiographic hip OA	1.130	0.009	3.094 (3.036–3.152)	<0.001
Constant	−5.850			<0.001

CI, confidence interval; sex, female; BMI, body mass index; occupation, having a blue-collar job; residence, living in rural area; depression, feeling depressed for more than 2 weeks; radiographic hip OA, radiological findings indicating more than moderate osteoarthritis of the hip based on the Kellgren–Lawrence grading system.

**Table 5:** Multivariate logistic regression analysis for knee pain in relation to diagnosis of cancer.

Characteristics	Coefficient	Standard Error	Odds Ratio (95% CI)	*p*-Value
Sex	0.699	0.005	2.012 (2.000–2.033)	<0.001
Age	0.044	0.0001	1.045 (1.045–1.045)	<0.001
BMI	0.053	0.0003	1.054 (1.053–1.055)	<0.001
Occupation	0.243	0.002	1.276 (1.271–1.281)	<0.001
Residence	0.233	0.002	1.250 (1.245–1.255)	<0.001
Menopause	0.233	0.005	1.262 (1.249–1.275)	<0.001
Depression	0.560	0.002	1.750 (1.742–1.758)	<0.001
Osteoporosis	0.513	0.002	1.670 (1.661–1.679)	<0.001
Gastric cancer	0.017	0.009	1.017 (0.997–1.037)	0.080
Liver cancer	0.093	0.021	1.098 (1.053–1.144)	<0.001
Colorectal cancer	−0.200	0.012	0.818 (0.797–0.838)	<0.001
Breast cancer	−0.303	0.001	0.738 (0.725–0.751)	<0.001
Cervical cancer	−0.081	0.011	0.921 (0.900–0.942)	<0.001
Lung cancer	−13.01	6.48	0.000002 (0–0.732)	<0.05
Thyroid cancer	−0.072	0.013	0.930 (0.911–0.949)	<0.001
Radiographic knee OA	0.950	0.002	2.586 (2.576–2.596)	<0.001
Constant	−6.698			<0.001

CI, confidence interval; sex, female; BMI, body mass index; occupation, blue-collar job; residence, living in rural area; depression, feeling depressed for more than 2 weeks; radiographic knee OA, radiological findings indicating more than moderate osteoarthritis of the knee based on the Kellgren–Lawrence grading system.

## Data Availability

All KNHANES dataset files are available at the KNHANES website: https://knhanes.kdca.go.kr/knhanes/eng/main.do, accessed on 21 January 2025.
